# Central Pontine Myelinolysis: A Case Report of Persistent Hyperglycemia With Normal Serum Sodium

**DOI:** 10.7759/cureus.29470

**Published:** 2022-09-22

**Authors:** Iwalewa G Olowoporoku, Lakshmi P Digala, Dinanath P Attele

**Affiliations:** 1 Neurology, University of Missouri, Columbia, USA; 2 Neurology, University of Missouri School of Medicine, Columbia, USA

**Keywords:** hyperosmolar hyperglycemic state, pseudobulbar affect, chronic, osmotic demyelination syndrome, susceptibility weighted imaging, restricted diffusion, microbleeds, normal serum sodium, hyperglycemia, cerebral fat embolism

## Abstract

Rapid correction of hyponatremia is the most frequent predisposing factor for the development of central pontine myelinolysis (CPM). Alcoholism, cirrhosis, malnutrition, and severe burns are associated conditions that often present in combination with a rapid rise in serum sodium concentration. However, its association with hyperglycemia has not been as well established. There have been recent reports of acute to subacute presentation of CPM with hyperglycemia. We report an unusual case of a 48-year-old Caucasian male who presented with pseudobulbar palsy, ataxia, and quadriplegia with worsening pontine hyperintensities and was diagnosed with CPM in the setting of persistent hyperglycemia with normal serum sodium.

## Introduction

Central pontine myelinolysis (CPM), a condition described in 1959 by Adams et al., was first associated with young alcoholics [1]. While hyponatremia is the major predisposing factor, conditions like hypokalemia, liver cirrhosis, severe burns, and acquired immunodeficiency syndrome have also been implicated in this condition [2]. Hyperosmolar states such as hyperglycemia are also known associations due to falsely decreased sodium levels with rebound hypernatremia following overcorrection [3]. However, there have been reports of hyperglycemia with no preceding hyponatremia [4]. Several timelines of CPM have been discussed, some of which are acute and subacute presentations [3,5]. In this case, we present a 48-year-old male with chronic presentation who was diagnosed with CPM due to persistent hyperglycemia.

## Case presentation

**Figure 1:**
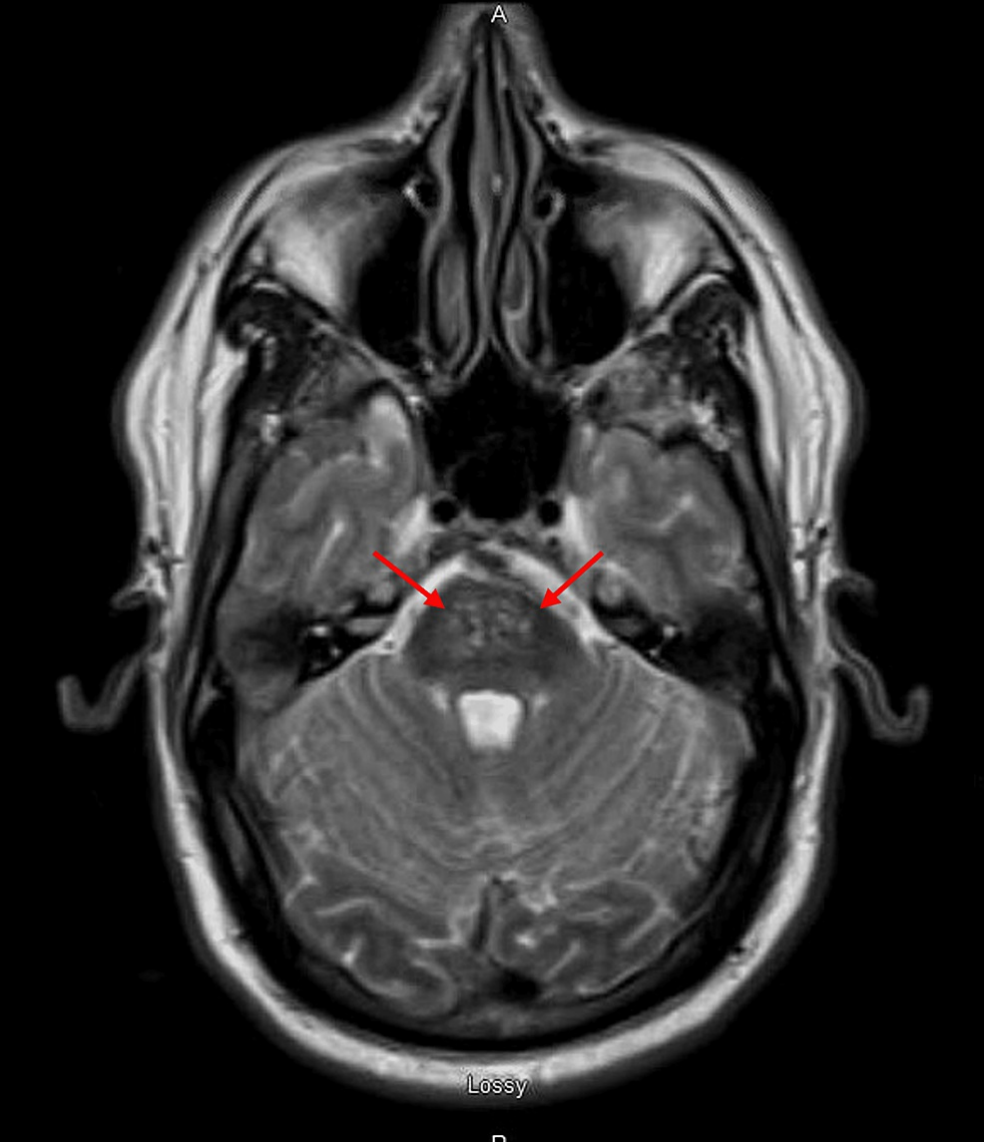
Arrows showing pontine hyperintensities in the initial T2-weighted imaging

A 48-year-old Caucasian male presented with generalized limb weakness and incoordination of about one-month duration. He also complained of excessive thirst and eating. At the time of presentation, no focal neurologic deficit was seen on examination. Blood glucose was 608 mg/dL with a corrected sodium level of 133 mmol/L and normal serum potassium of 3.8 mmol/L. His hyperglycemia was treated. Glycated hemoglobin was 14.7%. He had no prior history of diabetes mellitus before this presentation. He had magnetic resonance imaging (MRI) of the brain, which showed signal intensity in central pons on T2-weighted images (Figure 1). He was subsequently discharged home with an endocrinology follow-up and oral hypoglycemic agent. He was not compliant with his medication and was lost to follow-up.

Two months following his last hospital visit, he was seen in the emergency room where he complained of progressive symmetric generalized limb weakness for about four months, with slurred speech, labile emotions, and uncontrollable laughter for one month. On physical examination, he was noted to have pseudobulbar palsy, quadriparesis, which was worse in proximal muscle groups, diffuse hyperreflexia, fasciculations in the right lower extremity, extensor plantar response, decreased vibration in bilateral lower extremities, negative Romberg test, and ataxia. He was wheelchair-bound. Laboratory data revealed hyperglycemia (serum glucose: 391 mg/dL), normal serum sodium of 136 mmol/L, normal potassium of 3.7 mmol/L, and glycated hemoglobin of 12.4%. The initial working diagnosis was a motor neuron disease with the upper and lower motor neuron signs; however, electromyography was done, which showed no evidence of motor neuron disease, and a mild predominant sensory axonal length-dependent polyneuropathy was seen, which is likely due to his uncontrolled diabetes mellitus. The fasciculations were thought to be due to diabetic neuropathy.

**Figure 2:**
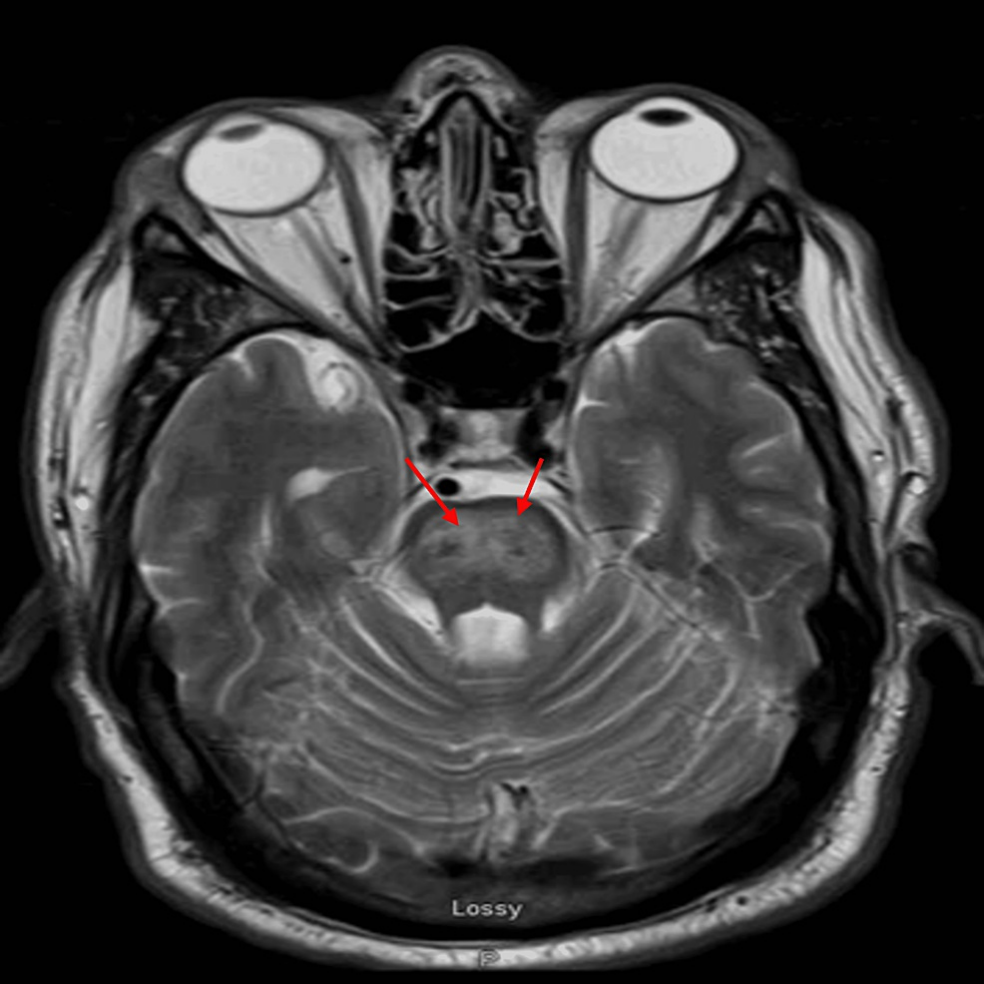
T2-weighted image showing worsening pontine hyperintensities on repeat imaging

**Figure 3:**
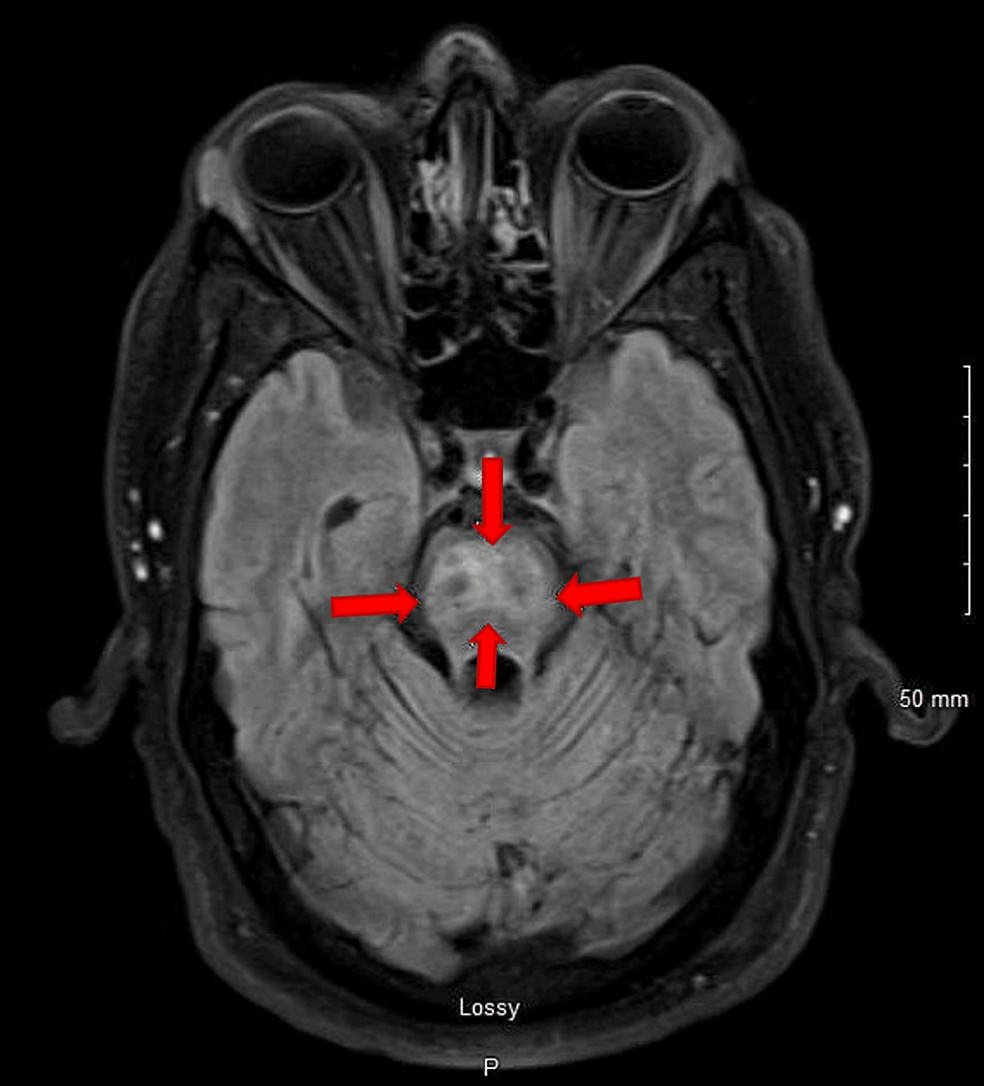
Arrows showing pontine hyperintensities on fluid-attenuated inversion recovery (FLAIR) sequence

MRI of the brain showed worsening signal intensity in the central pons on T2-weighted and fluid-attenuated inversion recovery (FLAIR) sequences (Figures 2, 3). There was also restricted diffusion in the central pons on magnetic resonance diffusion-weighted image (MR-DWI) with no apparent diffusion coefficient (ADC) correlation. At this point, we entertained the possibility of subacute stroke; however, the pattern and central location of the pons alongside no apparent diffusion correlate made a vascular etiology less likely. With the central pontine location of signal intensity and imaging characteristics (T2-weighted signal intensity with restricted diffusion on MR-DWI), he was diagnosed with CPM. His hyperglycemia was treated and he clinically improved; however, his ataxia persisted. He was subsequently discharged to in-patient rehabilitation. At his most recent clinic visit, he was able to ambulate unassisted and his glycated hemoglobin was 7%.

## Discussion

The term “central pontine myelinolysis” was first described by Adams in 1959 as a symmetric, demyelinating focus prominent in the central pons [1]. Before that, in 1950, a young alcoholic had presented with tetraplegia and pseudobulbar palsy. His postmortem diagnosis revealed a large size lesion that was characterized by demyelination, which was majorly in the basis pontis [2]. Chronic alcoholism has been mostly linked to CPM followed by rapid correction of hyponatremia and the third being in liver transplant patients particularly due to cyclosporine use [2]. Other conditions in which an association with CPM has been linked include malignant tumors of the lungs and gastrointestinal tract, diabetes mellitus, acute porphyria, pregnancy, change in immunological status, cerebral infarction, and chemotherapy [2].

In 1988, McKee et al. concluded based on postmortem diagnoses that extreme serum hyperosmolality contributed to CPM, which was due to hypernatremia, hyperglycemia, and azotemia, alone or combined [4]. Hyponatremia was not found in any of the patients in whom CPM was diagnosed and it was proposed that the rapid correction of hyponatremia exerts its effects by causing an osmotic shift and not because of specific properties of the sodium ion [4]. Several mechanisms have been described where rapid correction of sodium levels in chronic hyponatremia leads to shifts of potassium and other organic substances, which cannot be introduced to the cells as fast when rapid hyponatremia correction occurs and oligodendrocytes damage occurs as myelin sheath is stripped from the axon [2,6]. Also, Norenberg et al. proposed that the cerebral vascular endothelium is injured by a rapid rise in serum osmolality, and this results in the release of myelinotoxic factors and/or the production of vasogenic edema [7]. Others have demonstrated that a rapid rise in plasma osmolality can cause an osmotic opening of the blood-brain barrier [8] through transvesicular transport [8,9], which is usually transient.

The pons, which is primarily affected, was proposed to be due to an extensive gray-white matter admixture providing a suitable environment for interaction between the richly vascularized gray matter and myelin-containing white matter [7]. In 1982, Kleinschmidt-DeMasters et al. demonstrated extrapontine sites of myelinolysis in rats by causing vasopressin-induced hyponatremia with subsequent treatment with hypertonic saline [10]. Demyelination was seen in the corpus striatum, lateral hemispheric white matter, cerebral cortex, hippocampal fimbria, anterior commissure, thalamus, brainstem tegmentum, and cerebellum [10].

Despite all of the cases where CPM has been reported, there are fewer reports of subacute and chronic presentations of hyperglycemia [5]. In this case, the patient had a chronic presentation as the symptoms had been ongoing for at least three months. Saini et al. reported a case in 2015 where symptoms had been ongoing for two weeks and were initially mistaken for a posterior circulation stroke; however, the patient had hyperglycemia with normal corrected sodium, and with the treatment of hyperglycemia, the symptoms improved [5]. Many cases have been reported with hyperglycemia and pseudohyponatremia; however, it is important to also mention that CPM can occur with a hyperglycemic state despite normal corrected sodium [11], such as in this case. Some CPM cases have been described in the hyperglycemic hyperosmolar state with hypernatremia [12]. Also, a subacute to a chronic presentation can occur in persistently elevated serum osmolality where increased hyperintensity in the pons may be seen with repeat imaging.

Clinical features that have been described include quadriparesis, bulbar disorders, pathological reflexes, pseudobulbar emotionality, incontinence, decreased alertness, and confusion [13]. In this case, there was quadriparesis, pseudobulbar palsy, and pathological reflexes with sensory ataxia. The corticobulbar and corticospinal tract appeared to have been more susceptible to myelinolysis given his presentation. Microscopically, the lesions are characteristic as myelin surrounding the axons is typically destroyed and neurons of axons are well preserved. The involved area could have sharp borders and be irregular in appearance [13]. Cavitation can be seen in the center of the lesion in a few cases. Astrocytes are typically not altered but oligodendrocytes are either absent or degenerated [13]. Axonal dystrophy has been recorded in some patients [14]. The gross icteric discoloration seen in some patients indicated disruption of the blood-brain barrier [13].

Imaging findings of CPM include symmetric signal intensity abnormality in the central pons at T2-weighted and FLAIR imaging [15], which may progress to classic hyperintense “trident-shaped” [16], as seen in our case (Figures 2, 3). The Piglet sign, which is less known, has also been described [16]. The new lesions are symmetrical and hypointense on T1-weighted images in the acute state while subacute are hyperintense on T2-weighted images [17]. In some cases, there could be a clinico-radiological dissociation as MRI can lag behind the clinical picture by a few weeks [18]. This is because demyelinated patches do not show up until one or two weeks later [18], which could allow for delayed diagnosis. A repeat imaging in about 10-14 days may help support diagnosis if clinical suspicion already exists [18]. MR-DWI sequence, which is sensitive to water motion [19] and in turn osmotic shifts, has also been shown to be useful in diagnosis [7] and may be a better method to identify early pathophysiologic change given the underlying process of CPM [6]. This may also explain why some cases could be mistaken for vascular ischemia and misdiagnosis or delayed diagnosis may occur.

## Conclusions

While there have been several associations with CPM, such as alcoholism and rapid correction of hyponatremia, hyperglycemia is one of the less recognized associations. Recent evidence points to an association with hyperglycemia beyond pseudohyponatremia as the hyperosmolar state can also induce demyelination in the pons. Therefore, a diagnosis of CPM should be considered in diabetic patients with poor glycemic control.
